# Assessing Changes in Sodium Content of Selected Popular Commercially Processed and Restaurant Foods: Results from the USDA: CDC Sentinel Foods Surveillance Program

**DOI:** 10.3390/nu11081754

**Published:** 2019-07-30

**Authors:** Jaspreet K. C. Ahuja, Ying Li, David B. Haytowitz, Rahul Bahadur, Pamela R. Pehrsson, Mary E. Cogswell

**Affiliations:** 1 U.S. Department of Agriculture, Agricultural Research Service, Beltsville Human Nutrition Research Center, 10300 Baltimore Ave, Bldg 005, BARC-WEST, Beltsville, MD 20705, USA; 2 2968 Schubert Dr., Silver Spring, MD 20904, USA; 3 Centers for Disease Control and Prevention, Division for Heart Disease and Stroke Prevention, 4770 Buford Highway NE, Mail Stop F-73, Atlanta, GA 30341-3717, USA

**Keywords:** sodium, food composition, hypertension, sodium reduction, monitoring, variability, FDA sodium reduction targets

## Abstract

This report provides an update from the U.S. Department of Agriculture - Centers for Disease Control and Prevention Sentinel Foods Surveillance Program, exploring changes in sodium and related nutrients (energy, potassium, total and saturated fat, and total sugar) in popular, sodium-contributing, commercially processed and restaurant foods with added sodium. In 2010–2013, we obtained 3432 samples nationwide and chemically analyzed 1654 composites plus label information for 125 foods, to determine baseline laboratory and label sodium concentrations, respectively. In 2014–2017, we re-sampled and chemically analyzed 43 of the Sentinel Foods (1181 samples), tested for significant changes of at least ±10% (*p* < 0.05), in addition to tracking changes in labels for 108 Sentinel Foods. Our results show that the label sodium levels of a majority of the Sentinel Foods had not changed since baseline (~1/3rd of the products reported changes, with twice as many reductions as increases). Laboratory analyses of the 43 Sentinel Foods show that eight foods had significant changes (*p* < 0.05); sodium content continues to be high and variable, and there was no consistent pattern of changes in related nutrients. Comparisons of changes in labels and laboratory sodium shows consistency for 60% of the products, i.e., similar changes (or no changes) in laboratory and label sodium content. The data from this monitoring program may help public health officials to develop strategies to reduce and monitor sodium trends in the food supply.

## 1. Introduction

High sodium intake has been linked to increased chronic disease risk, especially for cardiovascular diseases [1,2,3]. Americans (>14 years of age) consume over 3400 mg/day on average [4], i.e., about 50% more sodium than the recommended limit of less than 2300 mg/day in the 2015–2020 Dietary Guidelines for Americans (DGA) [5], Healthy People 2020 [6], and the 2019 Dietary Reference Intake level recommended for Chronic Disease Risk Reduction [1]. The majority of the sodium intake in the U.S. is from commercially processed and prepared foods with added sodium [7,8]. Hence, national and international public health agencies consider reducing sodium content of these foods to be a cost-effective public health strategy [9,10,11]. The Institute of Medicine (IOM) in 2010 recommended gradual reduction in sodium content, mandatory targets for reducing sodium in these foods, and mechanisms to monitor sodium in the food supply [12]. The New York Salt Reduction Initiative (NSRI), a public-private collaboration, set voluntary sodium targets in 2009 for several packaged and restaurant food categories. Many food manufacturers and restaurant chains in the U.S. pledged to reduce sodium in their products [13,14,15,16,17]. In 2016, U.S. Food and Drug Administration (FDA) issued draft voluntary targets (short-term (2 year) and long-term (10 year)) for reducing sodium for 150 food categories, and estimated that their implementation would reduce sodium intakes to about 3000 mg/day over 2 years and about 2300 mg/day over 10 years [18].

In 2010, in response to the IOM recommendations to enhance monitoring and surveillance of sodium content of foods, Centers for Disease Control and Prevention (CDC) and the U.S. Department of Agriculture (USDA), initiated the Sentinel Foods Surveillance Program. Under this program, the Nutrient Data Laboratory (NDL) of USDA initiated tracking 125 Sentinel Foods using multiple means, to assess changes in their sodium contents. The list included foods such as cheddar cheese, white bread, macaroni and cheese, french fries, representing several food types and both packaged and prepared/restaurant food sources. These foods were sampled nationwide mainly in 2010–2013, and chemically analyzed in the laboratory for ‘baseline’ sodium contents [19]. In addition to sodium, the following nutrients were also tracked, as they are most likely to change as manufacturers reformulate—energy, potassium, total and saturated fat, and total sugar, henceforth referred to as ‘related nutrients’ [12]. The complete list of Sentinel Foods, the monitoring plan and its details have been published [20], and are summarized later in the ‘Methods’ section of the paper. Thereafter, NDL was to track the foods annually using information from food manufacturers such as manufacturer or restaurant websites, food labels etc. (referred to as labels hereafter), and resample the Sentinel Foods nationwide and chemically analyze them at periodic intervals.

The objective of this report is to provide an update of results from USDA-CDC Sentinel Foods Surveillance Program, exploring changes in sodium and related nutrients in selected commercially processed packaged and restaurant foods, using dual methods - food labels and laboratory analyses. We compared results from the two methods to provide insights into changes in the marketplace and the monitoring methodologies. Furthermore, we compared the resampled laboratory sodium values to the FDA’s draft voluntary sodium-reduction targets and sodium limits for ‘healthy’ foods (as done for baseline sodium contents [19]).

## 2. Methods

### 2.1. Selection of Sentinel Foods

The 125 Sentinel Foods were selected in 2010 to serve as indicators for assessing changes or temporal trends. They are comprised of commercially processed packaged foods (92 of 125) and prepared (fast food or restaurant) foods (33 of 125). Their selection was based on evaluation of sodium concentration (mg/100 g of food), frequency of consumption by respondents in the national survey (What We Eat In America (WWEIA), NHANES 2007–2008), and percentage of contribution to sodium intake, and accounted for approximately one-third of total sodium intake of the U.S. population, excluding breast-fed infants [20]. These foods continue to be popular as indicated in the latest survey (WWEIA, NHANES 2015–2016) [21]. The complete list of the 125 Sentinel Foods is available in Supplemental Table S1. The foods are grouped by food type, adapted from WWEIA Food Categories [22], for purposes of presenting the data.

### 2.2. Assessment of Baseline Sodium Content

**Scheme 1:**
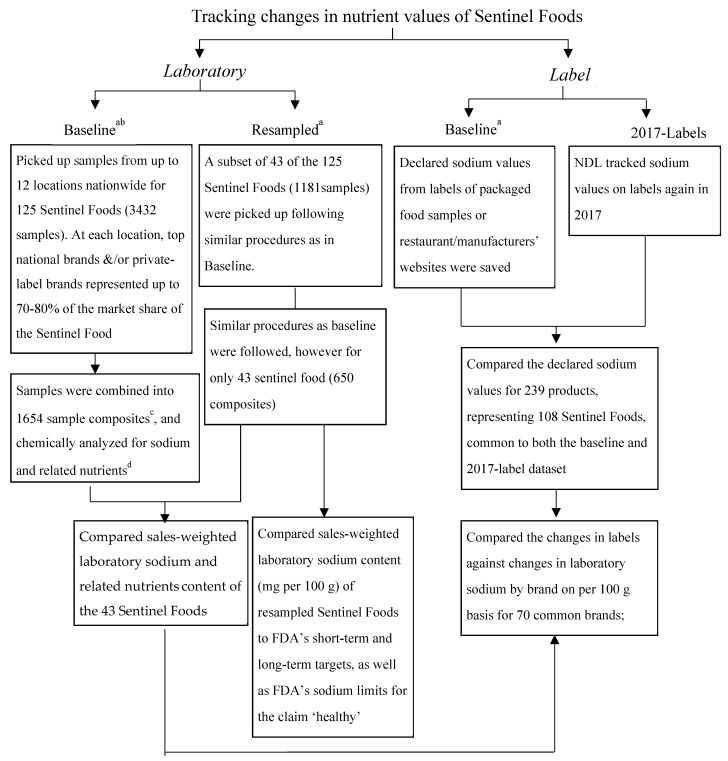
Summary of the steps, methods, and sample sizes used for tracking changes in nutrient values, as a flow chart. ^a^ Baseline Sentinel Foods were sampled in 2010–2013 and resampled in 2014–2017. ^b^ Please see Reference [20] for details on sampling plan and chemical analysis methodology. ^c^ Composite-homogenized one or more samples of the same food. ^d^ Related nutrients studied-energy, potassium, total and saturated fat, and total sugar.

NDL sampled and analyzed the Sentinel Foods staggered over the years 2010–2013 (supplemental sampling for three foods-canned corn, cheddar cheese and mozzarella cheese was done in 2014), using the protocols established under the National Food and Nutrient Analysis Program [23,24]. The salient features of the program include use of a statistically valid, 3-stage probability-proportional-to-size sampling plan, laboratory analysis of foods using valid, approved methods by pre-qualified laboratories, and multi-step quality control reviews by chemists and nutritionists. NDL developed a sampling plan for each Sentinel Food, using the most recent census data to identify cities and counties, store-based retail sales data to identify retail stores, and most recent consumer point-of-sale Nielsen data [25] to determine the market share of the brands, and the top brands to sample. Using the sampling plan, NDL purchased 3432 samples of the 125 Sentinel Foods from high-sales retail outlets at up to 12 locations nationwide (See Scheme 1). The samples included national and private-label (store brands) for the packaged foods. Prepared foods were sampled from major fast food, family-style restaurants and local restaurants, as appropriate. The sampling process and selection of brands and restaurants are detailed elsewhere [20].

The purchased samples were shipped to laboratories at Virginia Tech or Texas Tech Universities and prepared as per package directions (if needed). One or more samples, mostly of the same brand but two different locations, were further combined and homogenized into 1654 composites, to reduce cost of chemical analyses (See Scheme 1). These composites were shipped, along with blinded matrix-matched in-house or standard reference quality control materials to pre-qualified laboratories to measure sodium, potassium, total fat, saturated fat, total sugars, total nitrogen, moisture, ash, and alcohol (if applicable) concentrations per 100 g, among others. Not all composites were analyzed for all nutrients, to save on laboratory costs. For example, canned tuna and bacon were not analyzed for total sugar, and concentration was assumed to be zero. The methods of analysis and sample sizes for each nutrient/food component are listed in Supplemental Table S2. The results from chemical analysis were reviewed by chemists and nutritionists, and validated against the results from blinded reference materials [26]. NDL calculated carbohydrate by difference for each of the composites using the equation: Total carbohydrate by difference = 100 − [water, protein, total lipid, ash, and alcohol in g/100 g], and total energy based on the Atwater system [27]. The nutrient contents for sodium and related nutrients of the composites were then weighted by market share of the brands of the composites to determine nationally representative sales-weighted nutrient contents for the Sentinel Foods, using the following equation, sales-weighted mean = ∑*wx/*∑*w,* where w represents the market share of the brand of the composite, determined using point-of-sales data, and *x* refers to the analytical nutrient content of the composite.

Information on the nutrition facts panel (NFP) from packaged food samples or from restaurant websites, if available (henceforth referred to as labels), were saved for 110 Sentinel Foods. These generally included major national brands and 1–2 private-label brands. No labels were available for 15 foods, mainly from in-store bakery or local restaurants, such as fried rice from local Chinese restaurants. Multiple label values were saved when the sodium values were different for the same brand collected from different retail outlets. The nutrient values declared on *labels* and from *laboratory* analysis of samples mainly obtained from 2010–2013, represent the baseline nutrient values against which future assessments were compared to track changes.

### 2.3. Tracking Changes in Nutrient Values

The Scheme 1 summarizes the steps, methods, and sample sizes used for tracking changes in nutrient values.

Changes in *label* nutrient values: NDL tracked labels of Sentinel Foods again in 2017 (some labels were collected in late 2016), representing major national brands and selected private-label brands (generally Walmart^®^ or Safeway^®^ brands, if available). The brands and the type of product selected (e.g., Brand A crinkle fries for french fries, frozen) were based on the most recent consumer point-of-sale from Information Resources, Inc. (IRI) [28]. Sodium content declared on the label, and the source (directly from manufacturers or restaurant chains, their websites, or the NFP of the products) from where the information was obtained were saved in a Microsoft Excel spreadsheet. A post-hoc comparison of declared label sodium values against the analytical contents for the baseline samples, showed that the majority of the values were in agreement, supporting the use of labels as a tracking mechanism [29]. For this report, we compared the label serving weight and sodium values per serving for 108 Sentinel Foods, represented by 1–5 labels common to both the baseline and 2017-label dataset (239 products). Twenty-eight labels did not have the serving weight information listed, mainly restaurant foods; hence, we assumed the serving weight to be the same as at baseline. If there were changes in the serving size on the 2017-label, we used the 2017 serving weight to calculate sodium per 100 g (sodium per 100 g = (sodium per serving × 100)/serving weight). We determined changes in declared sodium values for each product label using the equation—change in *label* sodium (%) = (2017 *label* sodium − baseline *label* sodium)/(baseline *label* sodium) × 100, and grouped percent changes by brand into five categories—reductions in sodium values (<−20%; ≥−20% and ≤−10%), increase in sodium values (>20%; ≥10% and ≤20%), and no change (>−10% and <10%), and then determined their frequency distribution. A change of at least ±10% was considered as minimal change, as per our established criteria, based on our review of analytical variability for chemical analyses of sodium [20]. The comparisons were made for each product label, rather than Sentinel Foods, as different brands can potentially have changes in declared sodium content in different directions (i.e., reduce or increase or no change). We re-checked all changes over 20% against the label image by an alternate staff, to detect typographical errors.

Changes in *laboratory* nutrient contents*:* As per the initial monitoring plan, the 125 Sentinel Foods were to be periodically resampled and chemically analyzed every 4–8 years, and the periodicity was determined and described in detail [20]. Due to reduced funding, only a subset of 43 of the 125 foods were resampled. The initial plan was supplemented with several other data sources to identify the 43 Sentinel Foods. These data sources included—a. review of top 10 food categories for sodium intake in the U.S., to ensure adequate representation of the top categories [30]; b. dietary intake data from the latest WWEIA, NHANES, prioritizing Sentinel Foods with higher contribution to total sodium intake; c. changes in label sodium values prioritizing Sentinel Foods with changes ; d. Sentinel Foods with larger differences in label and laboratory values at baseline [29]. NDL resampled 43 Sentinel Foods staggered over years 2014–2017 (Table 1) (exception: white bread was sampled in late 2013, and packaged macaroni and cheese and ramen noodles were re-sampled in early 2018), to obtain 1181 samples, combined one or more samples into 650 composites and chemically analyzed them using similar procedures as in baseline, with one exception. Consumer point-of-sale data from Nielsen [25] used to identify brands to sample in baseline was replaced with similar data from IRI [28]. We determined the sales-weighted mean sodium content for each of the 43 resampled Sentinel Food and compared to their baseline sales-weighted mean content, and determined change using the equation: Change in *laboratory* sodium (%) = (resampled laboratory sodium content − baseline laboratory sodium content)/(baseline laboratory sodium content) × 100. Furthermore, for foods with at least ±10% change in laboratory sodium value, we identified changes in related nutrients (potassium, total fat, saturated fat, total calories, and total sugar per 100 g) of at least ±10%. We did not compare total fat for three foods—canned tomatoes, green beans, and tuna, as their laboratory total fat contents for both baseline and resampled foods were less than 1 g/100 g, and our review of results for reference quality control material have shown high analytical variability for such foods. The resampled laboratory data were used for further evaluations, detailed below, as they provide insights especially on the variability of the foods and help validate the changes in labels.

### 2.4. Comparison of Changes in Labels and Laboratory Sodium at Brand Level

NDL identified changes in label sodium values from baseline to 2017 for the 43 resampled foods by brand. For one food (Brand A for canned ravioli), there were two 2017-labels with different declared sodium labels, so we used the mean sodium per 100 g as the 2017-label sodium for the brand. For seven sentinel foods (11 brands), we used 2015 label data, as these foods were chemically analyzed in 2015. Furthermore, we determined mean changes in laboratory sodium content by brand, and the changes in laboratory sodium of the Sentinel Foods by brand (%) using the equation: (resampled laboratory sodium content of brand − baseline laboratory sodium content of brand)/(baseline laboratory sodium content) × 100. We compared the changes in labels against changes in laboratory sodium by brand on per 100 g basis for 70 common brands. Private-labels and few top brands that changed due to changes in market share were not included in these comparisons. We grouped the results as follows—1. No change in label or laboratory sodium content, 2. changes in label sodium but not laboratory sodium, 3. changes in laboratory sodium but not label sodium, 4. changes in both label and laboratory sodium content in the same direction, and 5. changes in label and laboratory sodium content in the opposite directions.

### 2.5. Comparison to FDA’s Draft Voluntary Sodium-Reduction Targets

NDL assigned FDA’s draft food categories to the 43 Sentinel Foods, based on description and source (packaged or restaurant) (Supplemental Table S3). For most foods, the assignment was direct, such as the Sentinel Food, ‘Italian Dressing’ was assigned to the FDA category ‘Salad dressing’. For few foods, substitutions were used. For example, Sentinel Food, ‘French fries, frozen’ was assigned to the FDA category, ‘Fried Potatoes without Toppings’, restaurant as sodium targets were not available for packaged frozen french fries. FDA draft categories are broader and represent many foods other than the linked Sentinel Food and FDA used food labels for packaged foods and menu data for restaurants mainly from 2010, adjusted by market share of the sales volume to determine the baseline sodium concentrations [18]. Each of the categories have target sodium concentrations indicating FDA’s sales-weighted goals for the food category, short (2-year) and long-term (10-year). We compared estimated sales-weighted *laboratory* sodium content (mg per 100 g) of resampled Sentinel Foods to FDA’s short-term and long-term target sodium contents by calculating percent differences and identified foods with percent difference ≥ 10%, i.e., higher than 10% of the FDA’s target sodium content. Percent difference = (sales-weighted *laboratory* sodium content − FDA target)/FDA target) × 100. To provide better understanding of the results, we also compared baseline sodium content of Sentinel Foods to FDA’s baseline and targets.

### 2.6. Comparison to FDA Sodium Limits for ‘Healthy’ Foods

NDL compared amounts of sodium per serving to the FDA limits for sodium for the nutrient content claim ‘healthy’, using the same methods as used for baseline (based on type of food and RACC (Reference Amounts Customarily Consumed) or serving size [19]). We used the baseline categorization of the Sentinel Foods as ‘individual’ or ‘meal-type/main dishes’ and serving size/RACC information, and compared the sodium contents to the FDA criteria 21 CFR §101.65(d) (2) (480 mg per serving or per RACC for individual foods with RACC > 30 g; 480 mg/50 g for individual foods with RACC ≤ 30 g; 600 mg/serving for meal-type/main dishes) [31]. FDA has several other criteria for the nutrient content claim ‘healthy’; however, comparisons were only made for the sodium criteria.

### 2.7. Statistical Analysis

For foods with at least ±10% change in laboratory sodium content, NDL tested for significance of difference (*alpha* = 0.05) using the R (version 3.4.1) [32] package EMMeans [33], after rank transforming nutrient data, fitting a ranked regression model with the ranked nutrient content as the dependent variable and the type (baseline or re-sampled) as the independent variable, and weighting by market share. Statistical tests were not done when data were skewed or multi-modal, extremely heteroscedastic, sample sizes *(n)* were insufficient (*n* < 6), or when the differences were lower than 10%. The *p*-values for each food, across all nutrients were then adjusted for multiple comparisons using the ‘holm’ correction procedure. We also determined measures of variability (standard deviation (SD), coefficient of variability (CV) (=SD/mean), and range for each Sentinel Food) for sodium and related nutrients. All statistical analyses were performed using R (version 3.4.1).

## 3. Results

### 3.1. Tracking Changes in Label Sodium Values

**Figure 1:**
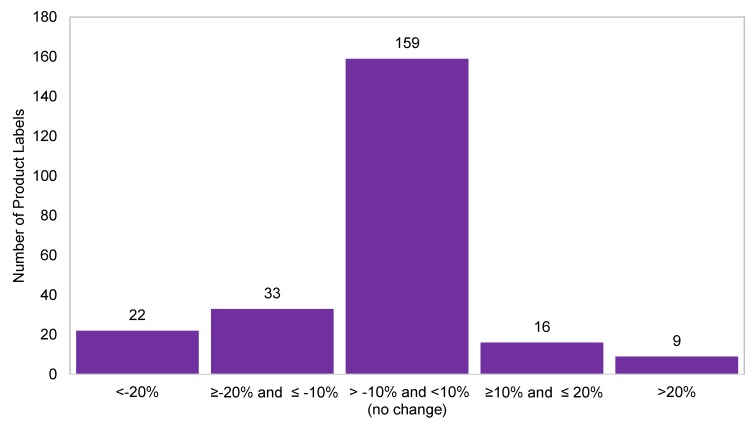
Distribution of changes (%). ^a^ in the *label* sodium values of 239 products representing 108 Sentinel Foods ^b^, from baseline to 2017. ^a^ Change in *label* sodium (%) = (2017 *label* sodium—baseline *label* sodium)/(baseline *label* sodium) × 100. Baseline labels were collected in 2010–2013. ^b^ Sentinel Foods: Popular, sodium-contributing, commercially processed and restaurant foods in the U.S. that are being tracked as part of the USDA-CDC sodium monitoring program [20].

Figure 1 presents a histogram of distribution of changes (%) in the declared label sodium values of 239 products (representing 108 Sentinel Foods) from baseline to 2017. The majority (66%) of the label sodium values did not change as per our criteria (at least ±10%). Changes were observed for about one-third of the labels (80 of 239 labels). There were more than twice as many reductions (*n* = 55 labels) as increases in declared sodium values (*n* = 25 labels).

Table 2 lists changes of at least ±10% in the label sodium values, between baseline and 2017, specific to brands, sorted by food category. Large changes (<−20% or >20%) in sodium content were observed for 31 of the 80 changes in labels, many of which were among top sodium contributors [30] including specific brands of American cheese, flour tortillas, pork sausage, fast food pepperoni and cheese pizza, and breaded chicken sandwich (reductions), whereas label sodium content increased for specific brands of American cheese, flour tortilla, white bread and hamburger rolls, bologna, meat and poultry frankfurters, among others. In terms of Sentinel Foods (not product labels, as in Figure 1), about half of the Sentinel Foods (*n* = 49 of 108) had no changes in label sodium levels for all brands examined. Sodium levels reduced for 39 foods and increased for 14 foods, consistently in the same direction for different brands of the same food. However, for six Sentinel foods-flour tortilla, fast food cheese pizza, fast food macaroni and cheese, frozen lasagna, American cheese and frozen corn dogs, some brands increased, whereas others decreased. Among the food categories represented by the 108 Sentinel Foods, there were no changes in label sodium levels since baseline among the category salad dressings and mayonnaise. Overall, changes in the sodium content of foods within categories were mixed, e.g., some foods in the cheese category decreased in sodium concentrations whereas others increased. The patterns of no change, reductions, and increases were similar irrespective of where the food was obtained from, i.e., store or restaurant. Of the 48 restaurant food labels tracked, 30 had no changes, 12 decreased, and six increased in sodium content. Similarly, for 191 packaged food labels tracked, 132 had no changes, 41 decreased, and 18 increased in sodium content. NDL tracked 37 labels for private-label products: 18 had no changes, 14 decreased, and five increased in sodium content.

### 3.2. Tracking Changes in Laboratory Nutrient Values

**Figure 2:**
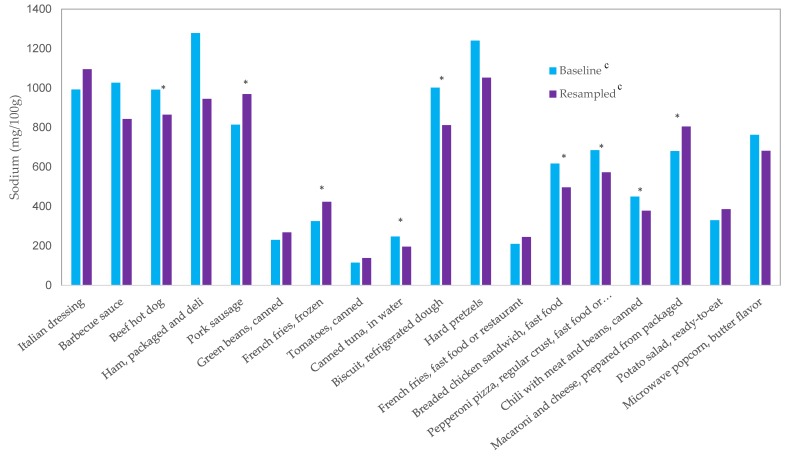
Sentinel Foods with changes ^a^ of at least ±10% in the sales-weighted mean *laboratory* sodium content (18 of the 43 resampled Sentinel Foods) ^b^, sorted by change in sodium content (mg per 100 g) *: *p* < 0.05. ^a^ Change in *laboratory* sodium (%) = (resampled ^c^ laboratory sodium content − baseline ^c^ laboratory sodium content)/(baseline laboratory sodium content) × 100. ^b^ Sentinel Foods: Popular, sodium-contributing, commercially processed and restaurant foods in the U.S. that are being tracked as part of the USDA’s sodium monitoring program[20]. ^c^ Baseline Sentinel Foods were sampled in 2010–2013, and then resampled in 2014–2017. * *p* < 0.05.

**Figure 3:**
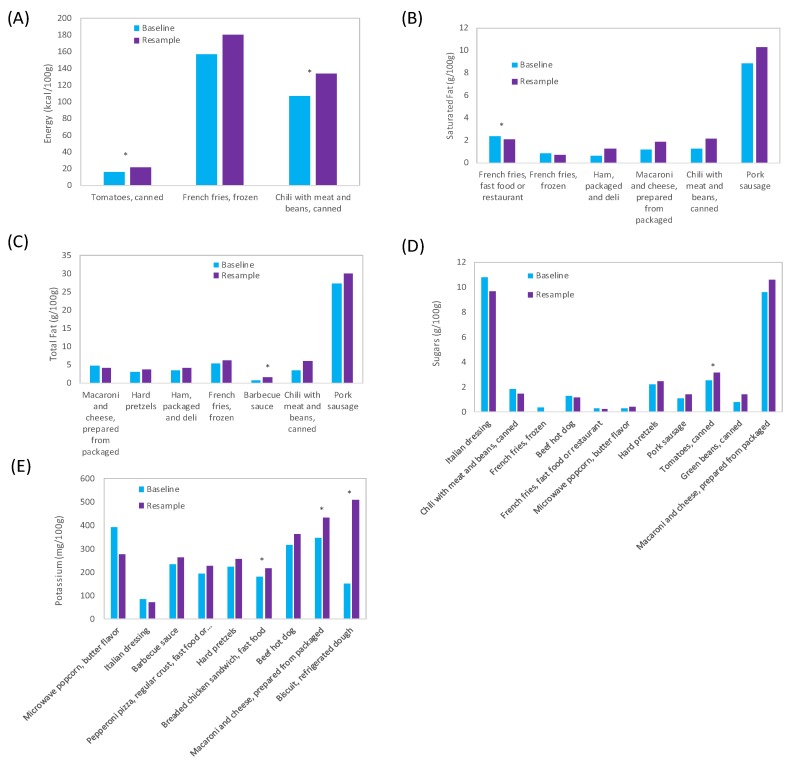
Resampled Sentinel Foods with changes of at least ±10% in sales-weighted sodium and related nutrients: (**A**) energy, (**B**) saturated fat, (**C**) total fat (excludes foods < 1 g per 100 g), (**D**) sugar, and (**E**) Potassium since baseline. Baseline Sentinel Foods were sampled in 2010–2013, and then resampled in 2014–2017. *: *p* < 0.05.

Table 3 lists baseline and resampled sales-weighted mean and variability measures for laboratory sodium content (mg per 100 g) and percent change in the sodium content. Our results show that for 25 of 43 Sentinel Foods resampled, sodium content did not change as per our criteria of at least ±10%. We observed changes for 18 of the 43 foods; for 10 of the 18 foods with changes, the sodium content reduced, whereas for the other eight foods the sodium content increased. These changes could have been due to market share changes or reformulation of products to reduce sodium. The changes were statistically significant for eight of the 43 foods (*p* < 0.05). The sodium concentration was 10% to 26% lower for beef hot dogs, canned tuna, refrigerated biscuit dough, fast food pepperoni pizza and canned chili with meat and beans, whereas the sodium concentration was 10% to 30% higher among pork sausage, frozen french fries, and packaged macaroni and cheese. Figure 2 presents the changes in sales-weighted mean baseline and resampled sodium content for these 18 foods sorted by relative change in sodium content. In terms of biggest absolute changes in mean sodium content, sliced ham decreased by 334 mg (26% decrease) from 1279 to 945 mg/100 g, and pork sausage increased by 155 mg (19% increase) from 814 to 969 mg/100 g. Figure 3 shows changes in related nutrient contents for 16 foods that had changes of at least ±10% for sales-weighted sodium and one or more related nutrient estimates. Of these 16 foods, there were significant changes (*p* < 0.05) for seven foods, as follows: increases in energy (two foods), total fat (one food), total sugar (one food), and potassium (three foods), and decrease in saturated fat for one food.

**Figure 4:**
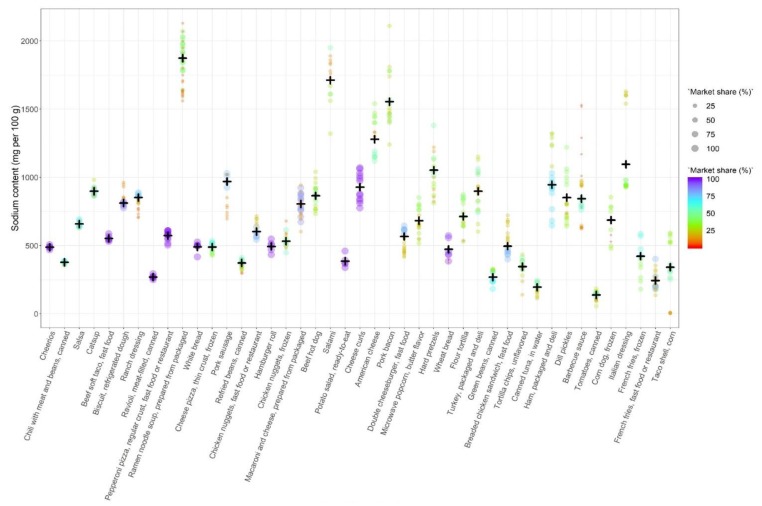
Variability of sodium per 100 g of the resampled Sentinel Foods (sampled in 2014–2017; *n* = 43 Sentinel Foods; 650 sample composites analyzed), sorted by variability (% CV). The size of the points is proportional to their market share. Color for the ease of the readers. The plus symbol indicates the sales-weighted mean sodium content (mg/100 g) for the food.

Figure 4 shows the results from laboratory analyses of sodium (mg/100 g) for individual composite samples (*n* = 650) for the 43 resampled foods. Taco shells, fast food and frozen french fries, Italian dressing, corn dogs, and canned tomatoes had the highest CVs. Cheerios, canned chili, salsa, catsup, and fast food soft beef tacos had the lowest CVs. Of these, for Cheerios and beef tacos only one prominent brand was sampled, whereas several brands were sampled for other foods. The range for sodium per 100 g was widest for barbecue sauce (623 to 1530 mg), pork bacon (1240 to 2110 mg), and Italian dressing (926 to 1630 mg) (all over 700 mg) among the 43 resampled foods.

Table 3 also compares the sales-weighted *laboratory* sodium content (mg per 100 g) of 43 resampled Sentinel Foods to FDA’s short-term and long-term target sodium concentrations for the category. Eighteen of the 43 resampled foods have a sodium content 10% or higher than the FDA short-term targets for their assigned category, nine of the 18 foods have mean sodium content ≥20% than the short-term target. The latter includes dill pickles, fast food soft beef tacos and pepperoni pizza, flour tortillas, frozen French fries, Italian dressing, potato salad, refried beans, and salsa. Twenty-five of the 43 resampled foods had sodium content either lower or within 10% of the FDA short-term target for the assigned category. Eighteen of these 25 foods had sodium content either lower or within 10% of the short-term FDA target at baseline too. The additional seven foods that meet the short-term targets on resampling are barbecue sauce, beef frankfurters, fast food chicken sandwich, microwave popcorn, packaged ham, pretzels, and refrigerated biscuits. However, four foods that had met the short-term FDA targets at baseline had increases in their sales-weighted sodium content, and do not meet the targets anymore. These include—corn dog, pork sausage, frozen french fries, and potato salad. Only four Sentinel Foods are either lower or 10% or less of the long-term FDA targets—canned green beans, tomatoes and tuna and taco shells.

Table 3 also compares the sales-weighted *laboratory* sodium content (mg per 100 g) of 43 resampled Sentinel Foods to FDA’s current sodium limits for the claim ‘healthy’. About half (21 of 43) of the Sentinel Foods, exceeded the FDA sodium limit for ‘healthy’, similar to baseline results. There were some shifts in the foods as compared to the baseline. There were increases in the mean sodium content per RACC for resampled pork sausage and potato salad exceeding the ‘healthy’ limit, whereas there were reductions in sodium content below the ‘healthy’ limit for refrigerated biscuit dough, barbecue sauce, beef frankfurters, and packaged ham. FDA guidelines require the food to meet several other criteria such as for fat and saturated fat content; however, we only compared to the sodium criteria. Hence, many of these foods may still not meet the FDA overall criteria for ‘healthy’.

**Figure 5:**
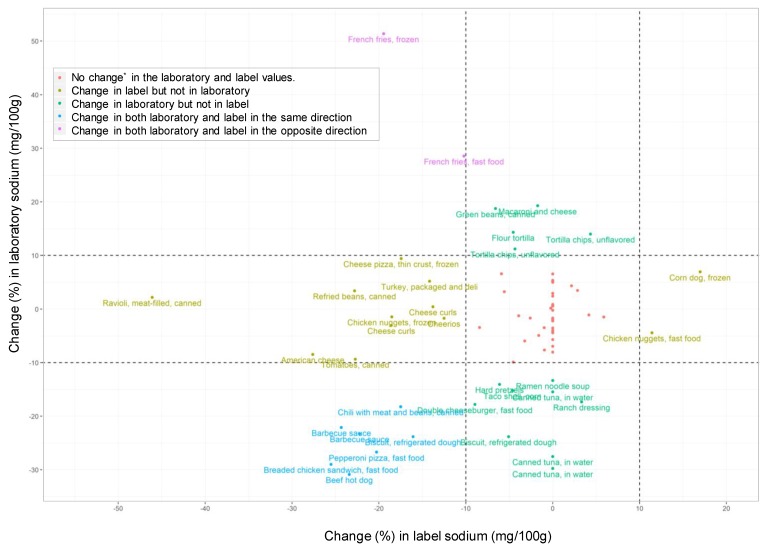
Comparison of changes (%) in the label and mean laboratory sodium (mg per 100 g) by brand (*n* = 70); * Change is defined as at least ±10% change in sodium content from baseline. To make the figure easy to read, product descriptions were shortened, and not added for products with no change in laboratory and label values (in red). Each observation represents a brand; hence, some descriptions may be repeated.

Figure 5 shows comparison of changes (%) in label and mean laboratory sodium by brand (mg per 100 g), for each of the 70 common brands. Our comparison shows the following-no change of at least ±10% in label or laboratory sodium content (*n* = 35 brands of the Sentinel Foods), changes in label sodium but not laboratory sodium (*n* = 12), changes in laboratory sodium but not label sodium (*n* = 14), changes in both label and laboratory sodium content in the same direction (*n* = 7), and changes in label (reduction) and laboratory sodium content (increase) in the opposite directions (*n* = 2).

## 4. Discussion

This report provides an update of results from the USDA-CDC Sentinel Foods Surveillance Program. Our results show that the *label* sodium levels of the majority of Sentinel Foods had not changed since baseline. About one-third of the products reported changes, with twice as many reductions as increases. Laboratory analyses of the Sentinel Foods and comparisons to the current FDA sodium criteria for ‘healthy’ and short- and long-term targets for sodium reduction shows that only eight of the 43 foods had significant changes (*p* < 0.05) and sodium content continues to be high and variable across brands and foods. Reductions in sodium were generally not accompanied by a consistent pattern of increase in calories, total fat, saturated fat, and sugar, or change in potassium. A comparison of changes by brand using dual methodology—labels and laboratory analyses—showed consistency for 60% of the products, i.e., similar changes (or no changes) in laboratory and label sodium content. For the rest, there were changes in labels, but not validated by laboratory analyses, and vice versa. The data from this program complement other sodium monitoring efforts at CDC and FDA [18,35], and may help public health officials to strategize methods and efforts needed to reduce and monitor sodium trends in the food supply.

Our results from tracking of *labels* are comparable to others [36]. Previous studies reported reductions in some categories but not all, and more reductions than increases in sodium content. However, these studies reported reductions in a much larger proportion of foods. For example, Clapp et al. [36] reported sodium reduction in about half of the products from 2009 to 2015, Poti et al. [37] for seven of the 10 food group sources of sodium from 2000 to 2014 by at least 10%, and Jacobson et al. [38] in ~40% of the ~400 products monitored from 2005 to 2011. We saw reductions in only a quarter of the products. This may be related to the different time periods and our definition of change as at least ±10%, among other factors. Clapp et al. defined it as ±1%, and others calculated it as any change in the mean value [36]. Jacobson et al. [38]) also noted an increase of 2.6% in mean sodium and increase in over half of the 78 fast food restaurant foods tracked from 2005 to 2011. Wolfson et al. [39] reported no significant changes (*p* < 0.05) in sodium content of foods from fast food/restaurants on the menu both in 2012 and 2016. We did not observe different patterns between packaged foods and foods from fast food/restaurants. Tracking restaurant foods using labels can have additional challenges, as the labeling guidelines for menu labeling do not require listing gram weights of the serving size on the labels, as is the rule for packaged foods [40]. Hence, changes in sodium content per serving may not translate to changes in sodium per 100 g.

The use of *laboratory* data provides several insights into the food environment. We continue to see substantial variability among different brands for a given food. For example, laboratory sodium content of taco shells ranged 3–593 mg/100 g (label values ranged from 0–571 mg/100 g (0–160 mg/serving)). The impact of variability on sodium intake is exacerbated when the serving sizes are higher. For example, the laboratory sodium values for different brands of fast food breaded chicken sandwich ranged from 400–721 mg/100 g. Therefore, a person who had one serving of the sandwich could potentially have a difference of ~500 mg per day i.e., a difference of about 20% of the daily value, depending on what brand they chose. The high variability among top brands in sodium contents highlights the potential for sodium reduction, as some brands have lowered the sodium content of their products, overcoming the technical challenges, while continuing to maintain high sales volume. Furthermore, it emphasizes the continuing need for consumer education for reading labels before purchase, as different brands of a similar food can have very different sodium content.

We also observed considerable variation in laboratory sodium content even within a brand. Differences of over 400 mg/100 g in sodium content within a brand were observed for specific brands of bacon, and ramen noodle soup; and on the basis of serving size, we observed differences of about 200 mg/serving (~7% of the Daily Value) for specific brands of fast food pepperoni pizza, fast food cheeseburger, and ramen noodle soup. It highlights the need for food manufacturers and fast food restaurants to improve control of the manufacturing process to reduce variability and improve accuracy of labels, as a strategy for sodium reduction [35].

While all Sentinel Foods are important contributors to sodium intakes in the U.S., a review of the foods that contribute the most represent the top 10 food categories in the U.S. [21], and were chemically analyzed in our study, shows mixed results—reductions (e.g., packaged ham, fast food pepperoni pizza, and breaded chicken sandwich), increases (e.g., pork sausage, packaged macaroni and cheese), and no changes (e.g., American cheese, ramen noodle soup, and white bread). Use of analytical data allows us to compare the data against older analytical sodium content data collected before baseline [20]. The analytic data prior to baseline shows that for some of the foods, such as white bread for which no changes in sodium concentration were observed from 2010, manufacturers might have made changes before our baseline study. However, the sales-weighted mean sodium content for some top contributors such as American cheese and ramen noodle soup remained unchanged.

Our review of changes in related nutrients does not show a pattern where food manufacturers and restaurants may increase total sugar, total fat and saturated fat when they reformulate their products to reduce their sodium content, as was a concern [12,20]. For example, of the 10 foods that had reductions in sodium, only four of them had significant (*p* < 0.05) changes of at least ±10% in sales-weighted related nutrient estimates. These were all increases-energy in canned chili, total fat in barbeque sauce, and potassium in barbecue sauce, refrigerated biscuit dough, and fast food breaded chicken sandwich. Some of changes may not be nutritionally significant, for example, the changes (*p* < 0.05) in total fat for barbecue sauce from 0.6 to 1.5 g/100 g. Changes in potassium were observed in about half of the foods with sodium changes, mainly increases, and three of the foods with sodium reductions. Some of these changes, although not all, could be related to addition of potassium salts (a salt substitute), such as in refrigerated biscuit dough. We did not observe the synergistic reductions in sodium and calories, as reported by Clapp et al. [36].

Comparisons to FDA’s proposed short-term and long-term targets at baseline and on resampling, and to the FDA’s sodium limit for the claim ‘healthy’ mimics the general pattern observed for sodium content of Sentinel Foods, i.e. no changes in majority, however, for foods with changes, there are more reductions than increases in sodium content. More than half of the Sentinel Foods had sodium content either lower or within 10% the FDA short-term target for the assigned category on resampling, similar to baseline. FDA categories are broader and represent many foods, for example, canned tomatoes assigned to FDA category ’canned vegetables’ also include Sentinel foods refried beans, and green beans, that have very different sodium profiles. Furthermore, FDA used label data mainly from 2010 or before to determine the baseline values, whereas the Sentinel Foods were mainly sampled in 2010–2013, and FDA regulations allow for over-declaration of sodium content [41]. Our objective was to identify foods that had sodium content either lower or within 10% the FDA targets, and not the exact target sodium content. For example, American cheese with sodium content of 1279 mg/100 g is within 10% of the FDA target, though higher than the target of 1210 mg/100 g. This method of using within 10% for comparison may not be similar to FDA’s methods of comparison. Irrespective, reductions in sodium content are needed for many foods to meet the short-term targets, and for almost all foods to meet the long-term targets. Similar to baseline, about half of the foods exceeded the sodium limit for the current FDA criteria for a “healthy” label claim at resampling, demonstrating the need for sodium reduction. At both baseline [19], and at re-sampling all main dishes and most restaurant foods exceeded the FDA sodium limit for the claim ‘healthy’, though only limited numbers were analyzed at resampling (5 main dishes and 6 restaurant items). 

Our use of dual methods to track changes-labels and laboratory analyses-provides perspectives not possible otherwise to the complexity of changes in the market place and the monitoring methodologies. Our comparison of changes in labels and laboratory analyses by brand (around similar time period and excluding private-label products), shows that 60% of the products either had no changes or changes in the same direction, based on both labels and laboratory analyses. For the rest, there were changes in labels, but not validated by laboratory analyses, and vice versa. For example, American cheese, brand A had a reduction in label sodium of 28%, but no changes in laboratory sodium values, whereas macaroni and cheese, brand A had no changes in the label sodium values, but the laboratory sodium content increased by 19%. Many foods, including canned tuna and ranch dressing (brand A) had no change in label values, but had a decrease in laboratory sodium, indicating possible stealth reduction in the product [41,42]. Only two products: fast food French fries, brand A and frozen French fries had label reductions but increases in laboratory sodium. A post-hoc analysis of the differences in baseline label and laboratory values for the Sentinel Foods showed majority of the label and laboratory sodium values were in agreement; about 19% of the label and laboratory values had differences of at least ±20% [31]. Overall, labels can provide a valid method for tracking sodium content, especially as nationwide sampling and chemical analysis is expensive and the U.S. food supply is constantly changing. Laboratory analyses are useful to validate changes in labels. Comparison of changes at the Sentinel Food level are more difficult to categorize and compare. It shows inconsistent reformulations across brands (in different directions or varying in level of change) and changes in market shares of the top brands, or combination of changes in product profiles and market shares. For some foods, substitutions of the UPC#’s were made for tracking labels or for collection of samples, further obscuring the situation.

Reducing the sodium content of commercially processed and restaurant foods has been recommended as an effective strategy to reduce sodium intake in the U.S. [12]. Reductions across the food supply are important, as it helps the consumers who may not read labels or are not aware of the need to reduce sodium. Cogswell et al. predicted a reduction of ~700 mg/day (3417 mg/day to 2719 mg/day) in the sodium intake of the U.S. population, aged 1 and older, if the sodium levels were to meet the NSRI targets [43]. The FDA draft long-term targets aim at reductions in sodium intake in the U.S., to ~2300 mg/day. Hence, continued reduction of sodium content of foods is necessary to achieve the population-wide reductions in dietary intake. Several food manufacturers have made consistent efforts and have been successful in reducing the sodium content of their products [44]. These efforts need to be continued, expanded, and accelerated along with monitoring, to ensure the efforts are sustained and any unintended consequences are averted.

### Strengths and Limitations

The strengths of our study include the selection of foods based on dietary intake data from the national survey, use of consistent methodology (statistically valid nationwide sampling, inclusion of major national and private-label brands, chemical analysis of foods using standardized analytical methods) and a two-prong approach (labels from samples, where possible plus chemical analysis, with intent to validate changes in labels) for determining sodium content of Sentinel Foods at baseline and resampling. Furthermore, laboratory analyses can provide information on nutrients not consistently provided on labels, such as potassium.

The limitations of the study include the limited number of Sentinel Foods being tracked (albeit contributing about one-third of the sodium intake, and representative of the top food sources), hence it does not provide a comprehensive review of the changes in the sodium content of foods in the market place in the U.S. The number of foods that were chemically analyzed was further reduced due to resource limitations, leading to selection bias in resampled foods. However, labels were reviewed for all Sentinel Foods, and majority of the labels were found to be accurate when tested at baseline [29]. While similar methodology was used at both time points, except for the change in the source of market-share data, there were instances when there were changes in the products sampled, due to non-availability of the desired products or purchase of different products but very similar front-of-package. Other limitations of the study methods have been previously detailed [19,20].

## 5. Conclusions

This report provides an update of results from the federal inter-agency Sentinel Foods Surveillance Program. Our results show that some progress had been made in sodium reduction in the market place; however, sodium content for many highly consumed foods continued to be high and variable. Continued efforts are needed by the food manufacturers to lower the sodium content of packaged and restaurant foods, and for public health officials to monitor the progress. Further, the study provides a better understanding of the complexity of sodium monitoring, which may help public health officials to strategize methods and efforts needed to reduce and monitor sodium trends in the food supply.

## Figures and Tables

Change in label sodium

**Table 1:** An alphabetical listing of the 43 Sentinel Foods ^a^ resampled in 2014–2017, sorted by food category ^b^.

Food Category	Sentinel Food Description
Breads, Rolls, Tortillas	
	Flour tortilla
	Hamburger roll
	Taco shell, corn
	Wheat bread
	White bread
Breakfast Cereals	
	Cheerios
Cheese	
	American cheese
Condiments and Sauces	
	Barbecue sauce
	Catsup
	Dill pickles
	Salsa
Cured Meats/Poultry	
	Beef hot dog
	Ham, packaged and deli
	Pork bacon
	Pork sausage
	Salami
	Turkey, packaged and deli
Grain Based Mixed Dishes	
	Macaroni and cheese, prepared from packaged
	Ravioli, meat-filled, canned
Meat and Poultry Mixed Dishes	
	Chili with meat and beans, canned
Mexican Mixed Dishes	
	Beef soft taco, fast food
Pizza	
	Cheese pizza, thin crust, frozen
	Pepperoni pizza, regular crust, fast food or restaurant
Plant-Based Protein Foods	
	Refried beans, canned
Potato Products	
	French fries, fast food or restaurant
	French fries, frozen
	Potato salad, ready-to-eat
Poultry Products	
	Chicken nuggets, fast food or restaurant
	Chicken nuggets, frozen
Quick Bread Products	
	Biscuit, refrigerated dough
Salad dressings and mayonnaise	
	Italian dressing
	Ranch dressing
Sandwiches	
	Breaded chicken sandwich, fast food
	Corn dog, frozen
	Double cheeseburger, fast food
Savory Snacks and Crackers	
	Cheese curls
	Hard pretzels
	Microwave popcorn, butter flavor
	Tortilla chips, unflavored
Seafood Products	
	Canned tuna, in water
Soups	
	Ramen noodle soup, prepared from packaged
Vegetable Products	
	Green beans, canned
	Tomatoes, canned

^a^ Sentinel Foods are 125 popular, sodium-contributing, commercially processed and restaurant foods in the U.S. that have sodium added during processing or preparation. These foods will serve as indicators to assess changes over time. ^b^ Adapted from What We Eat In America Food Categories [22].

**Table 2:** Changes of at least ±10% in the *label* sodium values of Sentinel Foods ^a^, specific to brands, sorted by food category ^b^ from baseline (2010–2013) to 2017.

Food Item	Baseline			2017-Label			
	Serving Weight (g)	Sodium Value (mg/Serving)	Sodium Value (mg/100 g)	Serving Weight (g)	Sodium Value (mg/Serving)	Sodium Value (mg/100 g)	Change from Baseline (%) ^c^
**Asian Mixed Dishes**							
Vegetable egg roll (Brand A) ^d^	85	420	494	71	390	549	11
Vegetable egg roll (Brand B)	87	410	471	87	460	529	12
Vegetable egg roll (Brand C)	89	410	461	86	460	535	16
**Breads, Rolls, Tortillas**							
Flour tortilla (Brand A)	120	300	250	43	160	372	49
Flour tortilla (Brand B)	49	440	898	45	320	711	−21
Hamburger roll ^e^	39	150	385	43	230	535	39
Hamburger roll ^e^	39	150	385	43	230	535	39
White bread	28	130	464	26	140	538	16
**Breakfast Cereals**							
Cheerios	28	160	571	28	140	500	−13
Frosted flakes	30	140	467	29	150	517	11
**Cheese**							
American cheese (Brand A)	19	250	1316	21	220	1048	−20
American cheese (Brand B)	21	270	1286	19	340	1789	39
Fried mozzarella sticks, fast food or restaurant		2640			2140		−19 *
Mozzarella cheese, part-skim	28	200	714	28	180	643	−10
Parmesan cheese, grated	5	85	1700	5	75	1500	−12
**Condiments and Sauces**							
Barbecue sauce (Brand A)	36	370	1028	36	280	778	−24
Barbecue sauce (Brand B)	36	450	1250	36	350	972	−22
Nacho cheese dip	34	280	824	30	340	1116	36
Cured Meats/Poultry							
Beef hot dog (Brand A)	57	550	965	57	480	842	−13
Beef hot dog (Brand B)	45	470	1044	42	360	857	−18
Bologna	28	280	1000	32	390	1219	22
Ham, packaged and deli	28	310	1107	56	500	893	−19
Meat and poultry hot dog	45	350	778	45	420	933	20
Pork sausage	68	710	1044	68	510	750	−28
Turkey, packaged and deli	51	460	902	28	210	750	−17
**Grain Based Mixed Dishes**							
Lasagna with meat, fast food or restaurant	421	2830	672		2070		−27 *
Lasagna with meat, frozen (Brand A)	227	860	379	227	980	432	14
Lasagna with meat, frozen (Brand B) ^e^	215	670	312	297	820	276	−11
Lasagna with meat, frozen (Brand B) ^e^	215	860	400	297	820	276	−31
Macaroni and cheese, fast food or restaurant (Brand A)	180	570	317		650		14 *
Macaroni and cheese, fast food or restaurant (Brand B)	236	1530	648		1220		−20 *
Ravioli, meat-filled, canned (Brand A) ^e^	252	700	278	425	750	176	−36
Ravioli, meat-filled, canned (Brand A) ^e^	252	950	377	425	750	176	−53
Ravioli, meat-filled, canned (Brand B)	254	1050	413	254	750	295	−29
Spaghetti with meatballs, canned	250	1050	420	255	750	294	−30
**Meat and Poultry Mixed Dishes**							
Chicken pot pie, frozen	234	800	342	200	750	375	10
Chicken pot pie, frozen (Brand A)	198	790	399	198	930	470	18
Chicken pot pie, frozen (Brand A)	283	1000	353	198	930	470	33
Chili with meat and beans, canned	247	1200	486	247	990	401	−18
Chili with meat and beans, fast food or restaurant		1010			1130		12 *
**Mexican Mixed Dishes**							
Bean burrito, fast food	198	960	485		1060		10 *
**Pizza**							
Cheese pizza, thick crust, fast food or restaurant	125	750	600	125	640	512	−15
Cheese pizza, thin crust, fast food or restaurant (Brand A)	76	350	461	88	460	523	14
Cheese pizza, thin crust, fast food or restaurant (Brand B)	89	800	899	89	610	685	−24
Cheese pizza, thin crust, frozen	146	890	610	149	750	503	−17
Pepperoni pizza, regular crust, fast food or restaurant	108	740	685		590		−20 *
Pepperoni pizza, thick crust, fast food or restaurant	123	910	740		700		−23 *
**Plant-Based Protein Foods**							
Peanuts, dry roasted, salted	28	170	607	28	150	536	−12
Refried beans, canned	120	570	475	120	440	367	−23
**Potato Products**							
French fries, fast food or restaurant	117	270	231	111	230	207	−10
French fries, frozen (Brand A)	84	360	429	84	290	345	−19
French fries, frozen (Brand B)	84	410	488	84	270	321	−34
Hash browns, fast food	168	810	482	169	1140	675	40
Tater tots, frozen	84	400	476	84	330	393	−18
**Poultry Products**							
Chicken nuggets, fast food or restaurant	60	350	583	60	390	650	11
Chicken nuggets, frozen	85	540	635	85	360	424	−33
Chicken tenders, fast food or restaurant	201	1320	657		940		−29 *
Chicken tenders, frozen	85	460	541	84	600	714	32
**Quick Bread Products**							
Biscuit, refrigerated dough	58	560	966	58	470	810	−16
Blueberry muffin	50	150	300	47	190	404	35
**Sandwiches**							
Breaded chicken sandwich, fast food	143	800	559	144	600	417	−26
Corn dog, frozen (Brand A)	75	540	720	75	470	627	−13
Corn dog, frozen (Brand B)	76	460	605	113	800	708	17
Hamburger, fast food	100	460	460	93	380	409	−11
**Savory Snacks and Crackers**							
Cheese curls (Brand A)	28	290	1036	28	250	893	−14
Cheese curls (Brand B)	28	350	1250	28	300	1071	−14
Cracker, saltine	16	150	938	16	135	844	−10
Microwave popcorn, butter flavor (Brand A)	33	390	1182	34	300	882	−25
Microwave popcorn, butter flavor (Brand B)	30	350	1167	32	300	938	−20
Potato chips, flavored	28	230	821	28	200	714	−13
Tortilla chips, flavored	28	250	893	28	210	750	−16
**Seafood Products**							
Canned tuna, in water	56	215	384	56	180	321	−16
Fish sticks, frozen	86	480	558	95	420	442	−21
Fried shrimp, fast food or restaurant		1270			590		−54 *
**Soups**							
Chicken broth, canned, ready to serve	245	970	396	245	860	351	−11
**Sweet Bakery Products**							
Chocolate sandwich cookie	34	160	471	34	130	382	−19
**Vegetable Products**							
Green beans, canned (Brand A)	116	400	345	121	320	264	−23
Green beans, canned (Brand B)	116	330	284	120	290	242	−15
Tomatoes, canned (Brand A)	121	220	182	121	170	140	−23
Tomatoes, canned (Brand B)	121	220	182	121	180	149	−18

^a^ Sentinel Foods are 125 popular, sodium-contributing, commercially processed and restaurant foods in the U.S. that have sodium added during processing or preparation. These foods will serve as indicators to assess changes over time. ^b^ Adapted from What We Eat In America Food Categories. ^c^ Change in *label* sodium (%) = (2017 *label* sodium—baseline *label* sodium)/(baseline *label* sodium) × 100; per 100 g or per serving (flagged with *). ^d^ Brand A, B etc. was added after description of the food item, if there was more than one brand with change of at least ±10%. ^e^ Two different labels from the same brand, with same sodium declaration, but different related nutrient value. * The comparison was based on per serving.

**Table 3:** *Laboratory* sodium content of resampled ^a^ Sentinel Foods ^b^ (sales-weighted), compared to baseline ^c^ sodium content (sales-weighted), FDA’s short- and long-term sodium targets, and FDA’s sodium limit for ‘healthy’ foods ^d^, sorted by food category ^e^.

Food Item	Baseline Sales-Weighted Sodium Mean (SD) ^f^	Resampled Sales-Weighted Sodium (mg per 100 g)				Change from Baseline (%)	FDA Targets (mg per 100 g)		Difference (%) between Resampled Laboratory Sodium and FDA Target ^h^	
		*n*	Mean (SD)	Range	CV (%) ^g^		Short-Term	Long-Term	Short-Term	Long-Term
**Breads, Rolls, Tortillas**										
Wheat bread	508(48)	10	472(68)	375–573	15	−7	420	300	13	58
Hamburger roll	499(34)	5	493(39)	433–548	8	−1	440	300	12	65
White bread	490(40)	6	490(34)	417–526	7	0	440	300	11	63
Flour tortilla	708(73)	16	712(105)	531–873	15	1	580	410	23	74
Taco shell, corn	323(168)	17	341(181)	3−593	53	5	490	320	−30	7
**Breakfast Cereals**										
Cheerios	496(0)	6	488(11)	468–507	2	−2	470	310	4	57
**Cheese**										
American cheese ^i^	1308(66)	16	1279(142)	1120–1540	11	−2	1210	1000	6	28
**Condiments and Sauces**										
Barbecue sauce	1026(292)	28	843(166)	623–1530	20	−18	900	760	−6	11
Salsa	710(37)	12	657(20)	626–692	3	−7	500	350	32	88
Catsup	906(37)	18	897(28)	860–981	3	−1	900	760	0	18
Dill pickles	809(218)	18	850(168)	643–1220	20	5	500	410	70	108
**Cured Meats/Poultry**										
Ham, packaged and deli	1278(240)	18	945(174)	647–1320	18	−26	1020	810	−7	17
Beef hot dog	992(109)	17	864(82)	732–1040	9	−13 *	900	730	−4	18
Pork bacon ^i^	1684(139)	18	1554(186)	1240–2110	12	−8	1700	1200	−8	30
Salami ^i^	1756(206)	13	1712(182)	1320–1950	11	−3	1630	1300	5	32
Turkey, packaged and deli ^i^	922(142)	18	897(154)	603–1150	17	−3	900	780	0	15
Pork sausage ^i^	813(103)	13	969(73)	697–1030	8	19 *	850	750	14	29
**Grain Based Mixed Dishes**										
Ravioli, meat-filled, canned ^i,j^	282(48)	6	269(13)	249–293	5	−5	330	220	−18	23
Macaroni and cheese, prepared from packaged ^i,j,k^	680(54)	24	804(74)	603–943	9	18 *	750	490	7	64
**Meat and Poultry Mixed Dishes**										
Chili with meat and beans, canned ^i,j^	449(20)	12	377(9)	354–388	3	−16 *	330	220	15	72
**Mexican Mixed Dishes**										
Beef soft taco, fast food ^i^	559(25)	6	552(18)	530–587	3	−1	390	220	42	151
**Pizza**										
Pepperoni pizza, regular crust, fast food or restaurant ^i,j^	684(75)	12	572(34)	503–610	6	−16 *	460	310	25	85
Cheese pizza, thin crust, frozen ^i^	471(14)	12	489(35)	428–535	7	4	420	260	16	88
**Plant-Based Protein Foods**										
Refried beans, canned ^i^	370(43)	18	373(28)	298–411	8	1	290	250	29	49
**Potato Products**										
French fries, fast food or restaurant	209(55)	18	243(72)	137–402	30	16	310	190	−21	28
Potato salad, ready-to-eat ^i^	329(72)	5	385(41)	338–460	11	17	300	140	29	176
French fries, frozen	324(48)	11	422(120)	179–586	28	30 *	310	190	36	123
**Poultry Products**										
Chicken nuggets, frozen ^i^	560(50)	15	529(47)	449–679	9	−5	570	420	−7	26
Chicken nuggets, fast food or restaurant ^i^	594(80)	12	602(47)	544–714	8	1	570	420	6	44
**Quick Bread Products**										
Biscuit, refrigerated dough	1002(101)	12	811(37)	774–962	5	−19 *	820	500	−1	62
**Salad dressings and mayonnaise**										
Ranch dressing	901(60)	18	851(39)	705–890	5	−6	880	590	−3	44
Italian dressing ^i^	992(42)	18	1095(275)	926–1630	25	10	880	590	25	86
**Sandwiches**										
Breaded chicken sandwich, fast food ^i^	616(32)	18	496(88)	400–721	18	−20	470	310	6	60
Corn dog, frozen^i^	668(87)	10	686(155)	479–853	23	3	610	400	13	72
Double cheeseburger, fast food ^i^	617(86)	18	566(74)	417–645	13	−8	480	300	18	89
**Savory Snacks and Crackers**										
Hard pretzels ^i^	1240(272)	17	1052(152)	811–1380	14	−15	1020	750	3	40
Microwave popcorn, butter flavor	762(163)	18	681(96)	516–851	14	−11	680	400	0	70
Cheese curls	942(120)	12	927(100)	775–1070	11	−2	870	550	7	69
Tortilla chips, unflavored	327(46)	16	346(62)	140–434	18	6	390	300	−11	15
**Seafood Products**										
Canned tuna, in water	246(58)	18	194(35)	112–242	18	−21 *	360	260	−46	−25
**Soups**										
Ramen noodle soup, prepared from packaged ^i,j,k^	1855(132)	44	1874(124)	1560–2130	7	1	1640	1290	14	45
**Vegetable Products**										
Green beans, canned	229(24)	12	268(46)	183–328	17	17	290	250	−8	7
Tomatoes, canned	115(37)	18	138(29)	56–184	21	20	290	250	−52	−45

^a^ Resampled: Resampled in 2014–2017. ^b^ Sentinel Foods are 125 popular, sodium-contributing, commercially processed and restaurant foods in the U.S. that have sodium added during processing or preparation. These foods will serve as indicators to assess changes over time. ^c^ Baseline: Sampled in 2010–2013. ^d^ FDA, U.S. Food and Drug Administration. FDA’s short- and long-term sodium targets: See Reference [18]. FDA’s sodium limit for ‘healthy’ foods: Title 21: Food and Drugs §101.65 Implied nutrient content claims and related label statements. See Reference [34]. ^e^ Adapted from What We Eat In America Food Categories [22]. ^f^ SD: standard deviation. ^g^ CV: coefficient of variation. ^h^ Percent difference = (sales-weighted *laboratory* sodium content-FDA target)/FDA target × 100. ^i^ Exceeds FDA limit for sodium for ‘healthy’ foods (480 mg/serving and /RACC for individual foods with Reference Amounts Commonly Consumed (RACC) >30 g; 480 mg/50 g for individual foods with RACC ≤ 30 g; 600 mg for meal-type/main dishes) [34]. ^j^ Assigned as a main dish for FDA sodium limit for ‘healthy’ foods comparison. ^k^ Dried or condensed forms were sampled and analyzed. The nutrient values are for the samples analyzed. However, sodium content of prepared form was used for comparison to FDA sodium limit for ‘healthy’ foods. * *p* < 0.05.

## Supplementary Materials

The following are available online at https://www.mdpi.com/2072-6643/11/8/1754/s1, Table S1: An alphabetical listing of the 125 Sentinel Foods, sorted by food category, Table S2: Methods used for chemical analysis and sample sizes for sodium and related nutrients at baseline and resampling, Table S3: Resampled Sentinel Foods (*n* = 43) and the corresponding FDA category for voluntary sodium reduction targets.
