# Supplementary material for: Assessing Changes in Sodium Content of Selected Popular Commercially Processed and Restaurant Foods: Results from the USDA: CDC Sentinel Foods Surveillance Program
Source: Nutrients. 2019 Jul 30;11(8):1754. doi: 10.3390/nu11081754 (PMC6723387; doi:10.3390/nu11081754)
Supplement: Supplementary file 1 [file nutrients-11-01754-s001.pdf]

**Supplemental Table S1**: An alphabetical listing of the 125 Sentinel Foods[a], sorted by food category[b].

| Food Category | Sentinel Food Description |
|---|---|
| Asian Mixed Dishes | |
| | Chicken and vegetables, Chinese restaurant |
| | Orange chicken, Chinese restaurant |
| | Vegetable egg roll |
| | Vegetable fried rice, Chinese restaurant |
| Breads, Rolls, Tortillas | |
| | Bagel |
| | Flour tortilla |
| | Hamburger roll |
| | Taco shell, corn |
| | Wheat bread |
| | White bread |
| Breakfast Cereals | |
| | Cheerios |
| | Frosted flakes |
| | Instant oatmeal, flavored |
| | Raisin Bran |
| Cheese | |
| | American cheese |
| | Cheddar cheese |
| | Cottage cheese, reduced or low fat |
| | Fried mozzarella sticks, fast food or restaurant |
| | Mozzarella cheese, part-skim |
| | Parmesan cheese, grated |
| Condiments and Sauces | |
| | Barbecue sauce |
| | Catsup |
| | Dill pickles |
| | Marinara sauce, ready to serve |
| | Nacho cheese dip |
| | Salsa |
| | Soy sauce |
| | Soy sauce, reduced sodium |

| | |
|---|---|
| | Tomato sauce, canned |
| Cured Meats/Poultry | |
| | Beef hot dog |
| | Bologna |

| Food Category | Sentinel Food Description |
|---|---|
| Cured Meats/Poultry | Ham, packaged and deli |
| | Kielbasa |
| | Meat and poultry hot dog |
| | Pork bacon |
| | Pork sausage |
| | Salami |
| | Turkey, packaged and deli |
| Grain Based Mixed Dishes | |
| | Lasagna with meat, fast food or restaurant |
| | Lasagna with meat, frozen |
| | Macaroni and cheese, fast food or restaurant |
| | Macaroni and cheese, prepared from packaged |
| | Ravioli, meat-filled, canned |
| | Spaghetti with meatballs, canned |
| | Spanish rice, fast food or restaurant |
| | Spanish rice, prepared from packaged |
| Meat and Poultry Mixed Dishes | |
| | Chicken pot pie, frozen |
| | Chili with meat and beans, canned |
| | Chili with meat and beans, fast food or restaurant |
| Meats | |
| | Pork chop |
| Mexican Mixed Dishes | |
| | Bean burrito, fast food |
| | Beef hard taco, fast food |
| | Beef soft taco, fast food |
| | Chicken soft taco, fast food |
| Pizza | |
| | Cheese pizza, thick crust, fast food or restaurant |
| | Cheese pizza, thin crust, fast food or restaurant |
| | Cheese pizza, thin crust, frozen |
| | Pepperoni pizza, regular crust, fast food or restaurant |
| | Pepperoni pizza, thick crust, fast food or restaurant |

| | |
|---|---|
| Plant-Based Protein Foods | |
| | Peanut butter |
| | Peanuts, dry roasted, salted |
| | Pork and beans, canned |
| | Refried beans, canned |
| | Refried beans, fast food or restaurant |
| Potato Products | |
| | French fries, fast food or restaurant |
| Food Category | Sentinel Food Description |
| | French fries, frozen |
| | Hash browns, fast food |
| | Mashed potato, fast food or restaurant |
| | Potato salad, ready-to-eat |
| | Tater tots, frozen |
| Poultry Products | |
| | Chicken nuggets, fast food or restaurant |
| | Chicken nuggets, frozen |
| | Chicken tenders, fast food or restaurant |
| | Chicken tenders, frozen |
| | Fried chicken thigh, fast food |
| | Fried chicken thigh, frozen |
| | Fried chicken wing, fast food |
| | Rotisserie chicken |
| Quick Bread Products | |
| | Biscuit, fast food |
| | Biscuit, refrigerated dough |
| | Blueberry muffin |
| | Cornbread, prepared from mix |
| | Pancakes, frozen |
| Salad dressings and mayonnaise | |
| | Italian dressing |
| | Mayonnaise |
| | Mayonnaise-type dressing |
| | Ranch dressing |

| Food Category | Sentinel Food Description |
| --- | --- |
| Sandwiches | |
| | Breaded chicken sandwich, fast food |
| | Breaded fish fillet, fast food or restaurant |
| | Corn dog, frozen |
| | Double cheeseburger, fast food |
| | Egg, cheese, and ham on muffin, fast food |
| | Fish sandwich with cheese, fast food |
| | Hamburger, fast food |
| Savory Snacks and Crackers | |
| | Cheese curls |
| | Cracker, Ritz-like |
| | Cracker, saltine |
| | Hard pretzels |
| | Microwave popcorn, butter flavor |
| | Potato chips, flavored |
| | Potato chips, unflavored |
| Savory Snacks and Crackers | Tortilla chips, flavored |
| | Tortilla chips, unflavored |
| Seafood Products | |
| | Canned tuna, in water |
| | Fish sticks, frozen |
| | Fried shrimp, fast food or restaurant |
| Soups | |
| | Chicken broth, canned, ready to serve |
| | Chicken noodle soup, prepared from canned, condensed |
| | Cream of mushroom soup, prepared from canned, condensed |
| | Ramen noodle soup, prepared from packaged |
| | Tomato soup, prepared from canned, condensed |
| | Vegetable soup, canned, ready to serve |

| | |
|---|---|
| Sweet Bakery Products | |
| | Chocolate cake with icing |
| | Chocolate chip cookie |
| | Chocolate sandwich cookie |
| | Cinnamon bun, packaged |
| | Frosted toaster pastry |
| | Plain cake doughnut |
| Vegetable Products | |
| | Coleslaw, fast food or restaurant |
| | Corn, canned |
| | Green beans, canned |
| | Tomato and vegetable juice |
| | Tomato juice |
| | Tomatoes, canned |

[a]Sentinel Foods are 125 popular, sodium-contributing, commercially processed and restaurant foods in the U.S. that have sodium added during processing or preparation. These foods will serve as indicators to assess changes over time [1].

[b]Adapted from What We Eat In America Food Categories [2].

**Supplemental Table S2**: Methods used for chemical analysis and sample sizes for sodium and related nutrients at baseline[a] and resampling[b]

| Nutrient | Methods | Numbers of composites (Resampled) | Numbers of composites (Baseline)[d] |
|---|---|---|---|
| Sodium and potassium | AOAC 985.01(3.2.06) and 984.27 (50.1.15) | 649 | 1,654 |
| Total fat | AOAC 922.06, 925.12, 989.05, or 954.02 | 650 | 1,615 |
| Sugars | AOAC 982.14 (32.2.07) | 508 | 675 |
| Saturated Fatty Acids | AOAC 996.06 (41.1.28A) | 459 | 907 |

[a]Baseline: 125 Sentinel Foods[e] sampled in 2010-2013.

[b]Resampled: 43 of the 125 Sentinel Foods were resampled in 2014-2017.

[c]Related nutrients studied - energy, potassium, total and saturated fat, and total sugar.

[d]For 125 Sentinel Foods [3].

[e]Sentinel Foods are 125 popular, sodium-contributing, commercially processed and restaurant foods in the U.S. that have sodium added during processing or preparation. These foods will serve as indicators to assess changes over time.

**Supplemental Table S3**: Resampled Sentinel Foods[a] (n=43) and the corresponding FDA category for voluntary sodium reduction targets [4]

| Sentinel Food | FDA Food Category Name | Packaged/Prepared |
|---|---|---|
| American cheese | Processed Cheese/Cheese Food (Semi-soft) | Packaged |
| Barbecue sauce | Condiments | Packaged |
| Beef hot dog | Frankfurters, Hot Dogs, and Bologna | Packaged |
| Beef soft taco, fast food | Tacos, Burritos, and Enchiladas | Prepared |
| Biscuit, refrigerated dough | Frozen Biscuits | Packaged |
| Breaded chicken sandwich, fast food | Poultry/Fish-based Sandwiches | Prepared |
| Canned tuna, in water | Canned Fish and Seafood | Packaged |
| Catsup | Condiments | Packaged |
| Cheerios | Ready-to-Eat Cereal, Puffed | Packaged |
| Cheese curls | Puffed Corn Snacks | Packaged |
| Cheese pizza, thin crust, frozen | Pizza: Without Meat/Poultry or Seafood - Frozen | Packaged |
| Chicken nuggets, fast food or restaurant | Reformed/Restructured, Breaded/Battered Chicken | Prepared |
| Chicken nuggets, frozen | Reformed/Restructured, Breaded/Battered Chicken | Packaged |
| Chili with meat and beans, canned | Canned Meals | Packaged |
| Corn dog, frozen | Hot Dogs on Buns and Corn Dogs | Packaged |
| Dill pickles | Pickled Vegetables | Packaged |
| Double cheeseburger, fast food | Hamburgers/Ground Meat Sandwiches: With Cheese | Prepared |
| Flour tortilla | Tortillas and Wraps | Packaged |
| French fries, fast food or restaurant | Fried Potatoes without Toppings | Prepared |
| French fries, frozen | Fried Potatoes without Toppings | Packaged |
| Green beans, canned | Canned Vegetables | Packaged |
| Ham, packaged and deli | Deli Meats - Ham | Packaged |
| Sentinel Food | FDA Food Category Name | Packaged/Prepared |
| Hamburger roll | White Bread | Packaged |
| Hard pretzels | Pretzels | Packaged |
| Italian dressing | Salad Dressing | Packaged |

| | | |
|---|---|---|
| Macaroni and cheese, prepared from packaged | Grain-based Meals/Entrees, Dry- Mix | Packaged |
| Microwave popcorn, butter flavor | Popcorn | Packaged |
| Pepperoni pizza, regular crust, fast food or restaurant | Pizza: With Meat/Poultry or Seafood - Not Frozen | Prepared |
| Pork bacon | Cooked Bacon | Packaged |
| Pork sausage | Precooked Sausage | Packaged |
| Potato salad, ready-to-eat | Potato Side Dishes | Packaged |
| Ramen noodle soup, prepared from packaged | Dry Mix Soup | Packaged |
| Ranch dressing | Salad Dressing | Packaged |
| Ravioli, meat-filled, canned | Canned Meals | Packaged |
| Refried beans, canned | Canned Vegetables | Packaged |
| Salami | Salami and Pepperoni | Packaged |
| Salsa | Vegetable/fruit-based Dips | Packaged |
| Taco shell, corn | Hard Taco Shells | Packaged |
| Tomatoes, canned | Canned Vegetables | Packaged |
| Tortilla chips, unflavored | Unflavored Grain Chips | Packaged |
| Turkey, packaged and deli | Deli Meats -Turkey/Chicken | Packaged |
| Wheat bread | Wheat and Mixed Grain Bread | Packaged |
| White bread | White Bread | Packaged |

[a]Sentinel Foods are 125 popular, sodium-contributing, commercially processed and restaurant foods in the U.S. that have sodium added during processing or preparation. These foods will serve as indicators to assess changes over time.

References

1. Ahuja, J.K.; Pehrsson, P.R.; Haytowitz, D.B.; Wasswa-Kintu, S.; Nickle, M.; Showell, B.; Thomas, R.; Roseland, J.; Williams, J.; Khan, M. Sodium monitoring in commercially processed and restaurant foods. *Am J Clin Nutr* **2015**, *101*, 622-631, Available online: https://www.ncbi.nlm.nih.gov/pmc/articles/PMC4501259/ (accessed on 9 May 2019).
2. U.S. Department of Agriculture. What We Eat in America: Food categories 2011-2012. Availabe online: https://www.ars.usda.gov/ARSUserFiles/80400530/pdf/1314/food_category_list.pdf (accessed on March 27, 2019).
3. Ahuja, J.K.; Wasswa-Kintu, S.; Haytowitz, D.B.; Daniel, M.; Thomas, R.; Showell, B.; Nickle, M.; Roseland, J.M.; Gunn, J.; Cogswell, M. Sodium content of popular commercially processed and restaurant foods in the United States. *Prev Med Rep* **2015**, *2*, 962-967, Available online: https://www.sciencedirect.com/science/article/pii/S2211335515001606 (accessed on 9 May 2019).
4. US Food and Drug Administration. Draft guidance for industry: voluntary sodium reduction goals: target mean and upper bound concentrations for sodium in commercially processed, packaged, and prepared foods. Availabe online: https://www.fda.gov/Food/GuidanceRegulation/GuidanceDocumentsRegulatoryInformation/ucm494732.htm (accessed on March 27, 2019).
